# Angiotensin I-Converting Enzyme Mutation (Trp1197Stop) Causes a Dramatic Increase in Blood ACE

**DOI:** 10.1371/journal.pone.0008282

**Published:** 2009-12-14

**Authors:** Andrew B. Nesterovitch, Kyle D. Hogarth, Vyacheslav A. Adarichev, Elena I. Vinokour, David E. Schwartz, Julian Solway, Sergei M. Danilov

**Affiliations:** 1 Department of Anesthesiology, University of Illinois at Chicago, Chicago, Illinois, United States of America; 2 Department of Medicine, University of Chicago, Chicago, Illinois, United States of America; 3 Department of Medicine, Albert Einstein College of Medicine, Bronx, New York, United States of America; 4 National Cardiology Research Center, Moscow, Russia; Roswell Park Cancer Institute, United States of America

## Abstract

**Background:**

Angiotensin-converting enzyme (ACE) metabolizes many peptides and plays a key role in blood pressure regulation and vascular remodeling. Elevated ACE levels may be associated with an increased risk for different cardiovascular or respiratory diseases, including asthma. Previously, a molecular mechanism underlying a 5-fold familial increase of blood ACE was discovered: Pro1199Leu substitution enhanced the cleavage-secretion process. Carriers of this mutation were Caucasians from Europe (mostly Dutch) or had European roots.

**Methodology/Principal Findings:**

We have found a family of African-American descent whose affected members' blood ACE level was increased 13-fold over normal. In affected family members, codon TGG coding for Trp1197 was substituted in one allele by TGA (stop codon). As a result, half of ACE expressed in these individuals had a length of 1196 amino acids and lacked a transmembrane anchor. This ACE mutant is not trafficked to the cell membrane and is directly secreted out of cells; this mechanism apparently accounts for the high serum ACE level seen in affected individuals. A haplotype of the mutant ACE allele was determined based on 12 polymorphisms, which may help to identify other carriers of this mutation. Some but not all carriers of this mutation demonstrated airflow obstruction, and some but not all have hypertension.

**Conclusions/Significance:**

We have identified a novel Trp1197Stop mutation that results in dramatic elevation of serum ACE. Since blood ACE elevation is often taken as a marker of disease activity (sarcoidosis and Gaucher diseases), it is important for clinicians and medical scientists to be aware of alternative genetic causes of elevated blood ACE that are not apparently linked to disease.

## Introduction

Angiotensin I-converting enzyme (ACE, CD143) is a Zn^2+^ carboxydipeptidase which plays key roles in the regulation of blood pressure and in the development of vascular pathology and remodeling [1]–[3]. ACE is constitutively expressed on the surface of endothelial cells, different absorptive epithelial and neuroepithelial cells [4]–[7], and cells of the immune system (macrophages, dendritic cells) [8]–[9]. Somatic ACE contains two catalytic centers in N- and C-terminal domains [10]), whereas a short testis-specific isoform expressed in germ cells contains an identical C-domain and only one catalytic center [11]–[12]. ACE was assigned as a common differentiation marker - CD143 [7], [13].

Besides membrane-bound forms of ACE, blood and other biological fluids contain a variable amount of soluble ACE. Serum ACE originates likely from endothelial cells [14] by proteolytic cleavage [15]–[16]. Soluble ACE from CHO cells transfected with human somatic ACE cDNA and porcine somatic ACE have C-termini consistent with cleavage of the Arg1203-Ser1204 peptide bond in a stalk region near the transmembrane domain [17]. The cleavage/secretion process is catalyzed by an unidentified membrane-bound secretase [18]. Seminal fluid ACE, whose concentration is 50-fold more than that of ACE in blood, likely originates from epithelial cells of the epididymis, which express an abundant amount of somatic ACE [19]. In healthy individuals, the level of ACE in the blood is very stable [20], whereas granulomatous diseases (sarcoidosis in particular) and Gaucher's disease lead to a significant increase of ACE activity in the blood [21]–[24]. Serial serum ACE measurements now are an essential tool for the diagnosis and monitoring the clinical course of sarcoidosis [25]–[27].

Recently, a mutation in the stalk region of ACE – Pro1199Leu [28]–[29] – explained a dramatic (5-fold) increase in ACE activity in the blood of affected individuals from Holland [28], Germany [30], and USA [31]. Despite the fact that people with this mutation exhibit no clinical abnormalities [28], testing for this mutation is of considerable clinical importance. For example, failure to appreciate that elevation of ACE level is genetically determined in an affected individual may lead to false diagnosis of neurosarcoidosis and consequently to unnecessary long-term immunosuppressive treatment [30] or diagnostic procedures [32].

Here we report the identification of a new mutation in ACE, namely, introduction of a stop codon after N1196, which leads to immediate secretion of the ACE molecule rather than retention on the cell membrane, resulting in a dramatic increase in ACE level in the blood. Interestingly, highly elevated level of ACE in affected individuals (more than 13-fold that in the normal population) was found in some individuals with airflow obstruction or hypertension.

## Materials and Methods

### Study Participants

The study was approved by the Institutional Review Boards of the University of Illinois at Chicago and the University of Chicago. Ten volunteers without sarcoidosis were chosen as control subjects for a study of sarcoidosis unrelated to this report. One volunteer (subject N1) was found to have serum ACE activity higher than 400 mU/ml (normal range with Hip-His-Leu as a substrate was 8–60 mU/ml - ref. [33]). First- and second-degree relatives of subject N1 were asked to participate in the present study to analyze whether N1's ACE mutation was associated with any clinical phenotype. After giving written informed consent (or for children, assent with parental/guardian permission), they were interviewed and spirometry was performed. Blood was taken from adults for determination of ACE activity in serum or plasma and for genotyping. Saliva was obtained from children for genotyping.

### ACE Activity Assay

ACE activity in human serum or plasma was measured using a fluorimetric assay with two ACE substrates (5 mM Hip-His-Leu or 2 mM Z-Phe-His-Leu) [34]–[35]. Briefly, 20–40 µl aliquots of serum or plasma, diluted 1/5–1/80 in PBS-BSA (0.1 mg/ml), were added to 200 µl of ACE substrate and incubated for the appropriate time at 37°C. The His-Leu product was quantified fluorometrically.

### Immunological Characterization of the Mutant ACE (Plate Immunoprecipitation Assay)

96-well plates (Corning, Corning, NY) were coated with anti-ACE mAbs via goat anti-mouse IgG (Pierce, Rockford, IL) bridge [33] and incubated with serum/plasma samples or soluble ACE secreted from CHO cells transfected with wild type or mutant ACEs, which were equilibrated for ACE activity with Hip-His-Leu as a substrate. After washing of unbound ACE, plate-bound ACE activity was measured by adding a substrate for ACE (Hip-His-Leu) directly into wells [33], [36].

### Sequencing and Genotyping

Genomic DNA was obtained either from whole blood (adults) or saliva (children). The whole ACE gene (∼24 Kb) was sequenced in two individuals; genomic DNA was isolated from index subject N1 and from patient DR, who has the Pro1199Leu mutation (DNA kindly provided by Dr. R. Stein, Penobscot Bay Medical Center, Rockport, ME) used as a positive control.

After the discovery of the mutation Trp1197Stop, genotyping of subjects N1 and DR was performed by another PCR-based restriction fragment length polymorphism assessment. The 292 bp DNA fragment was amplified by PCR with primers Ex25_292Fw (TCCGCACGGAGAACGA, exon 25) and Ex25_292Rv (CCTGCTGCGCATCCA, exon 26) using genomic DNA from subjects N1, N3 (mother of N1), N2 (father of N1) and DR (an unrelated individual with Pro1199Leu mutation). The restriction endonuclease BsaHI (AcyI) cuts this 292 bp PCR product from individual with native, wild-type ACE (N3) into two fragments of 235 bp and 57 bp. Subject DR with Pro1199Leu substitution has mutated the last nucleotide in the recognition sequence of BsaHI (AcyI): GACGCT* vs. GRCGYC. Subject N1 (Trp1197Stop) has mutation of the first nucleotide in the recognition sequence of this endonuclease: *AACGCC vs. GRCGYC. As a result, these two mutations (G-to-A mutation in the position 3699 (leading to Trp1197Stop substitution and C-to-T mutation at position 3705 - leading to Pro1199Leu substitution) both eliminate the BsaHI (Acy I) restriction site. Thus, restriction analysis with BsaHI can distinguish an individual with these two mutations from individuals with normal ACE, but cannot distinguish between these two mutations - Pro1199Leu (DR) and Trp1197Stop (N1 and N2).

However, the restriction endonuclease BsrI cuts the 292 bp PCR product from individuals with wild-type ACE (e.g., N3) in the same place as endonuclease BsaHI (AcyI) also creating two fragments of 235 bp and 57 bp. In contrast, mutation Trp1197Stop disturbs the recognition sequence of BsrI: ACTGA*A vs. ACTGGN. At the same time, mutation P1199L has no effect on BsrI digestion. Thus, restriction analysis with BsrI can distinguish individuals with mutation Trp1197Stop (N1, N2) from individuals with mutation Pro1199Leu (e.g., DR), but cannot distinguish between mutation Pro1199Leu and native ACE (N3). Both restrictases BsaHI (AcyI) and BsrI must be used to differentiate mutations Trp1197Stop and Pro1199Leu and native ACE.

Heterozygous reading was obtained from genomic DNA sequence of the patient N1 (∼24 Kb) in the SNPs rs4331 (exon 15), rs4353 (exon 20), rs4359 (intron 23), rs4362 (exon 24) and rs4363 (intron 25).

### Cladistic Analysis

To study similarities between the ACE haplotype of subject N1 and haplotype clusters (clades) that had been already found in human population, we used a computational method for minimizing the weighted sum of squares of the differences between observed and expected pairwise distances between samples and construction of the most parsimonious classification trees using routines DNAPARS and DRAWGRAM, programs included in the software package PHYLIP v3.67 [37]. Genomic DNA from N1 patient was sequenced, and homozygous positions in N1 were aligned with haplotypes of published clades.

### Site-Directed Mutagenesis and In Vitro Analysis of the Mutant ACEs

cDNAs encoding two mutant ACE proteins were created by: 1) mutation of the Trp (TGG) codon at position 1197 to a stop codon (TGA); 2) mutation of the Pro (CCG) codon at position 1199 to a Leu (CTG) codon, in pACE-wt, an expression vector (based on pcDNA3.1+/Hygro (Invitrogen Corp., Carlsbad, CA) containing the full-length somatic ACE cDNA controlled by CMV early promoter [39]. An oligonucleotide-directed mutagenesis system (Quick Change site-directed mutagenesis kit, Stratagene, La Jolla, CA) was used according to the manufacturer's recommendation.

The following oligonucleotides containing the desired mutations were used:

5′-CCGCAGTACAACTGAACGCCGAACTCCGC-3′ (sense) and 5′-GCGGAGTTCGGCGTTCAGTTGTACTGCGG-3′ (antisense) for the Trp1197Stop mutation; 5′-GTACAACTGGACGCTGAACTCCGCTCGCT-3′ (sense) and 5′-AGCGAGCGGAGTTCAGCGTCCAGTTGTAC-3′ (antisense) for the Pro1199Leu mutation.

Plasmid DNA was sequenced and clones with desired mutation were selected for each mutation.

Plasmids carrying the coding sequence for wild-type ACE and above mutants were expressed in CHO cells using Plus Reagent (Invitrogen Corp., Carlsbad, CA) for transient transfection and generation of stable cell lines. CHO cell lines expressing WT ACE and WTΔ (Gln1230X) have been previously described ([38] and [16], respectively). Culture medium (Ultra-CHO medium, Cambrex Bio-Science, Walkersville, MD) from these cells was used as a source of the secreted (soluble) ACE (wild type and mutants) for biochemical and immunological characterization.

### ACE Purification from Heparinized Plasma

ACE was isolated from heparinized plasma of individuals with normal level of ACE, from subject N1, and from patients having the Pro1199Leu mutation [28] using affinity chromatography on a Lisinopril-Sepharose column. Briefly, plasma (5–45 ml) diluted in HEPES (20 mM pH 7.5, NaCl 150 mM) was incubated with 5 ml of Lisinopril-Sepharose and after intensive washing of unbound proteins, ACE was eluted with 50 mM borate buffer, pH 9.5.

### Western Blot Analysis of Mutant ACEs

All samples for SDS electrophoresis were equilibrated to a final ACE activity of 200 mU/ml (Hip-His-Leu as a substrate) and were run using gradient (4–15%) Tris-HCl pre-cast SDS PAGE gels (Bio-Red Laboratories, Hercules, CA). After electrophoretic transfer of proteins to microporous PVDF-Plus membranes, each membrane was incubated in 10 mM Tris-HCl (pH 8.0) buffer containing 150 mM NaCl, 0.05% Tween 20, and 5% dry milk prior to incubation with hybridoma culture fluids overnight at 4°C. Subsequent steps were carried out with the biotin/streptavidin system (Amresco, Solon, Ohio) and peroxidase activity was developed using WestPico Super Signal Chemiluminescense substrate (Pierce, Rockford, IL).

## Results

### Discovery of a Novel ACE Mutation that Leads to High Serum ACE Activity

**Figure 1:**
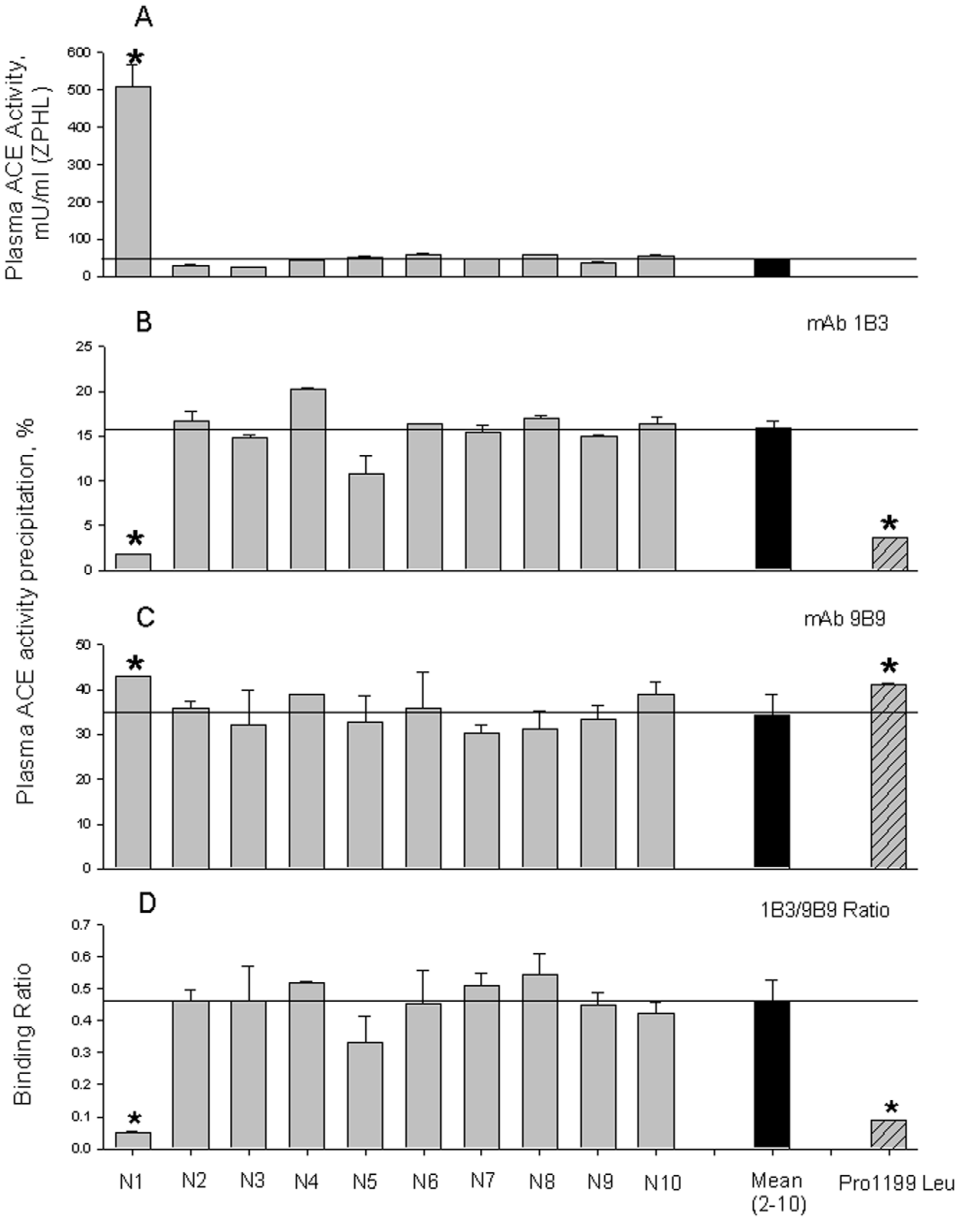
ACE activity and conformation in the heparinized human plasma. **A**. ACE activity in 10 samples of heparinized human plasma from healthy volunteers was quantified using a spectrofluorometric assay with Hip-His-Leu (5 mM) and Z-Phe-His-Leu (2 mM) as substrates. Samples were diluted 1/5 (N2-N10) and 1/80 ( N1). Data expressed as mU/ml (with ZPHL as a substrate) of whole plasma. The results are shown as means + SD of several (3–4) experiments. * - p<0.05 in comparison with mean value for samples N2-N10. **B–D**. Precipitation of ACE activity from plasma samples by mAbs 1B3 and 9B9. Heparinized plasma samples from of 10 volunteers were equilibrated according to 5 mU/ml of the ACE activity with Hip-His-Leu as a substrate and incubated with a wells on the microtiter plate covered by mAbs 1B3 or 9B9 via goat-anti-mouse IgG; then precipitated ACE activity was quantified by fluorimetric assay - plate precipitation assay [36], [39]. Data are expressed as a percentage of ACE activity precipitation by mAb 1B3 (**B**), mAb 9B9 (**C**) and as their ratio (**D**). Data are mean ± SD of triplicates. As a positive control sample of pooled plasma sample from several carriers of Pro1199Leu mutation of ACE [28], was used. * - p<0.05 in comparison with mean value for samples N2-N10.

During determination of ACE activity in 10 volunteers without sarcoidosis (participating as control subjects in a study of blood ACE activity in sarcoidosis), we found one individual (female, African-American, designated “subject N1”) with extremely high ACE activity – >13-fold greater than the mean ACE activity in the other volunteers (Fig. 1A). Recently, we reported a new assay that employs monoclonal antibodiy 1B3, recognizing an epitope on the stalk region of ACE where the Pro1199Leu (P1199L) mutation occurs, that identifies with high selectivity and specificity the mutation in the stalk region, and thus distinguishes hereditary elevation of ACE from that due to diseases [39]. We used this assay to analyze subject N1's blood ACE. Based on the immunoprecipitation of ACE by mAb 1B3 (Fig. 1B–D), ACE from subject N1 seems to have a mutation in the stalk region of ACE that is similar, but not identical, to that of individuals having Pro1199Leu mutation. Note that the 1B3/B9 ratio for ACE from subject N1 was about half that of patients having the P1199L mutation (Fig. 1D).

### Immunological Characterization of the Novel ACE from Subject N1

**Figure 2:**
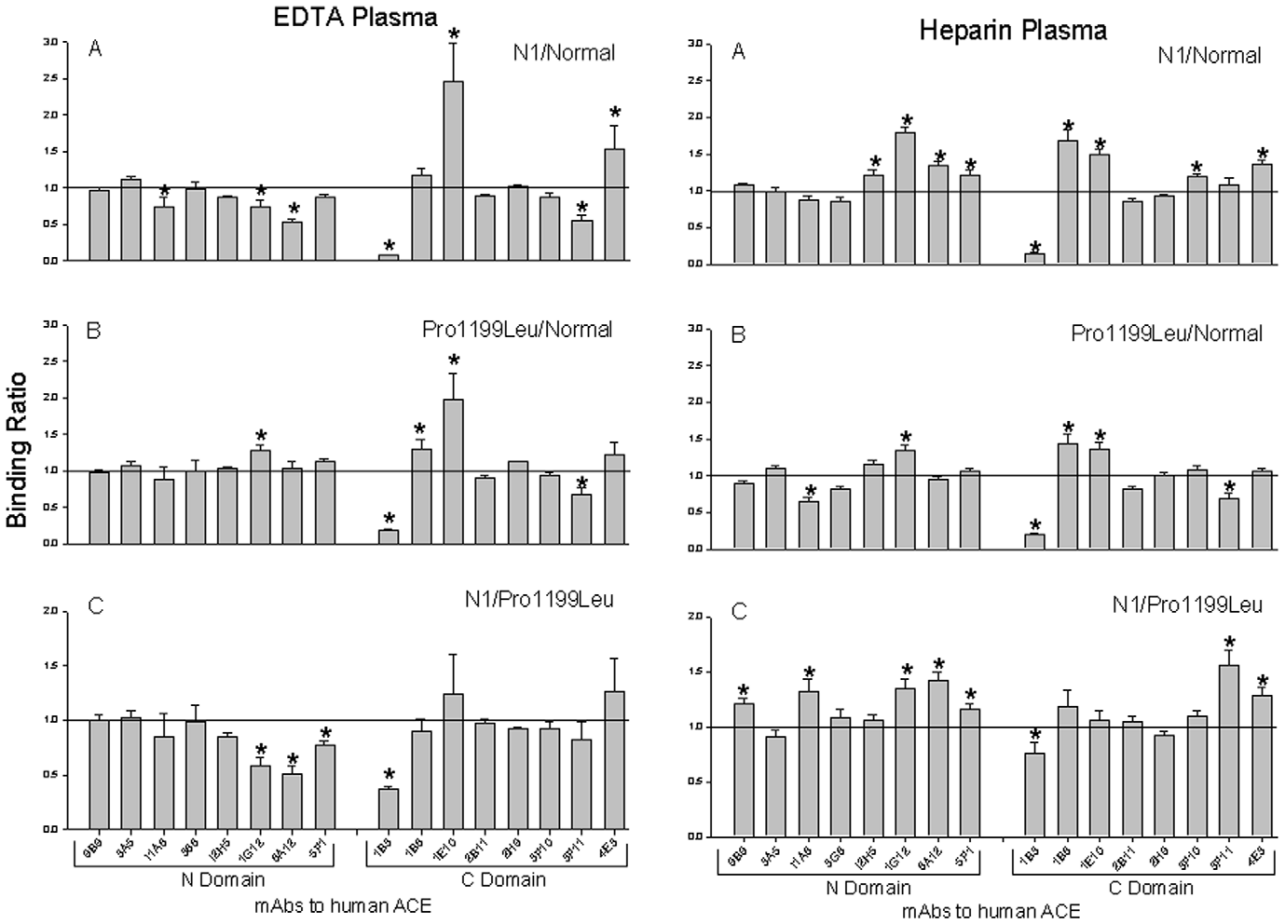
Conformational fingerprinting of mutant ACEs with a set of mAbs to ACE. Sixteen monoclonal antibodies were used to precipitate ACE from plasma anticoagulated using EDTA or heparin as indicated. Immunoprecipitated ACE activity is presented as a normalized value (“binding ratio”), to highlight differences in immunoprecipitation pattern (“conformational fingerprint”) among ACE variants from healthy volunteers (“Normal”), from subject N1 (“N1”), or carriers of the Pro1199Leu mutation (“Pro1199Leu”). (**A**) ACE binding ratios observed for N1 normalized to normal individuals; (**B**) ACE binding ratios observed for subjects with known Pro1199Leu mutation normalized to normal individuals; (**C**) ratio of ACE precipitation from subject N1 to that from carriers of Pro1199Leu mutation. Data presented as a mean of 6–8 independent determinations. * p<0.05 indicates ratio shown is significantly different from 1. These ratio for any pair of samples with normal ACE or with any pair of samples with one (Trp1197Stop) or another (Pro1199Leu) identical mutation was around 1.0 with no more than 10% of standard deviation (not shown).

The result above indicates that the mutant ACE from subject N1 is not immunologically identical to a previously known mutant ACE nor to wt ACE. We therefore further characterized the conformation of subject N1's mutant ACE, using a panel of mAbs directed against 16 different epitopes located on the N-and C-domain of catalytically active human ACE - “**conformational fingerprint of ACE**” (Danilov et al., manuscript in preparation). As apparent in Fig. 2, the immunoprecipitation profiles of ACE from subject N1 and of mutant P1199L ACE are similar but not identical, and both differ dramatically from that of ACE from other healthy volunteers. Note also that mutant Pro1199Leu ACE is differentially immunoprecipitated by several mAbs vs wt ACE (Fig. 2), and not only by mAb 1B3 as initially demonstrated [39]. It is also important to observe that the conformational fingerprinting of plasma ACE is quite different when plasma is collected from heparinized vs. EDTA-anticoagulated blood, which might be explained by the fact that depletion of Zn^2+^ from the active centers of N and C domains of ACE significantly changes a local conformation of ACE molecule [40].

### Sequencing of the ACE Gene of Subject N1

**Figure 3:**
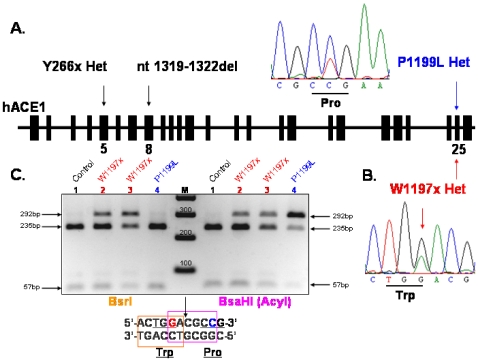
Organization of the identified mutations in ACE gene. Diagram shows intron-exon structure of human ACE1 gene and localization of the ACE1 mutations. **A**. Y266x Het mutation in 5^th^ exon and nt1319–1322del TGGA Hom mutation in 8^th^ exon were described in [54] and Pro1199Leu Het mutation in 25^th^ exon – in [28]–[29]. **B**. Heterozygous mutation Trp1197Stop (W1197x) in 25^th^ exon was revealed by the sequencing of the whole ACE gene (∼24 kb) and confirmed by the restriction analysis of the 292 bp PCR product flanking the part of exon 25 and intron 25 with restriction endonucleases BsrI and BsaHI (AcyI) (**C**), which was performed with genomic DNA of healthy individual with normal ACE (mother of N1) (1), patient N1 (2) and father of N1 (3) caring mutation Trp1197Stop, and carrier of Pro1199Leu mutation (4). Trp1197Stop (but not Pro1199Leu) mutation abolished restriction site for BsrI.

**Figure 4:**
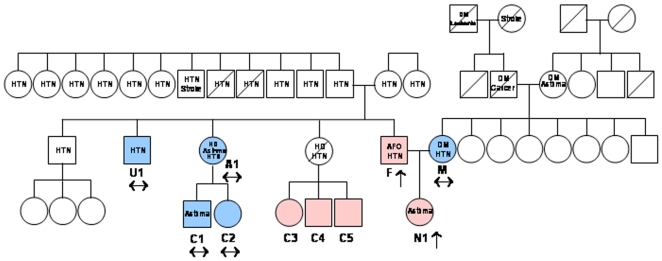
Family tree of subject N1 with new (Trp1197Stop) mutation in ACE gene. Individuals with Trp1197Stop mutation are indicated by red color. Genotyped individuals without Trp1197Stop mutation marked by blue color. Individuals found to have high level of serum ACE are marked by upward pointing arrows. Individuals found to have normal level of serum ACE are marked by horizontal arrows. Following abbreviations are used for known clinical diagnoses: HTN – hypertension, DM – diabetes mellitus, HD – heart disease, AFO – airflow obstruction, Asthma – asthma. ACE level in the blood of children (C3–C5) was not tested due to ethical considerations. Among individuals in whom both genotyping and serum ACE determination was performed, there was 100% concordance between presence or absence of the Trp1197Stop mutation and elevated or non-elevated serum ACE levels, respectively.

The novel conformational fingerprint of ACE from subject N1 (Figs. 1–2) suggested that N1 carries a novel ACE mutation located close to Pro1199, within the epitope for mAb 1B3. We therefore sequenced the ACE gene from subject N1 using genomic DNA, and as a control for sequencing we also sequenced ACE gene from a patient known to have the P1199L substitution. Sequence analysis of the genomic DNA from subject N1 disclosed a G to A mutation at position 3699 (nucleotide and amino acid numbering according to [10]), which leads to the replacement of a codon TGG for Tryptophan residue by a stop codon (TGA) at position 1197 in the stalk region of the mature ACE protein (Fig. 3B); subject N1 was heterozygous for this mutation. Moreover, sequence analysis of the complete coding region of the ACE gene in this individual revealed that beside other known polymorphisms that do not affect ACE levels, the G-A mutation at position 3699 is the only mutation present in the coding region of the ACE gene. As a result, half of ACE expressed in this patient had a length of 1196 amino acids and lacked the transmembrane anchor. Previous studies show that an ACE mutant (wtΔ), that lacks the transmembrane anchor-is not trafficked to the cell membrane and is directly secreted into the circulation [16]. We presume that a similar mechanism accounts for this subject N1's high serum ACE level. The Trp1197Stop (W1197X) mutation was not present in family members with normal ACE levels (Fig. 4). The hyper-ACE segregation pattern was compatible with autosomal dominant inheritance (Fig. 4).

The mAb 1B3-based method (Figs. 1–2) allows us to identify carriers of the novel W1197X mutation as well as P1199L substitution, but do not allow us to distinguish between these two mutations easily. To facilitate detection of the novel mutation, we used restriction analysis of a PCR-amplified fragment of genomic DNA spanning 292 bp including part of exon 25, intron 25, and part of exon 26. Fig. 3C demonstrates that the restriction endonucleases BsaHI (AcyI) and BsrI each cut the 292 bp PCR product from an individual with wild-type ACE (Control-mother of N1, Lane1) into two fragments of 235 bp and 57 bp. Both the G3678A mutation (leading to W1197X; subjects N1-Lane 2, and father of N1 –lane 3) and the C3683T mutation (leading to P1199L-Lane 4) eliminate the BsaHI (AcyI) restriction site. Therefore, restriction analysis with BsaHI (AcyI) can distinguish individuals with either of these two mutations from individuals with wt ACE, but cannot distinguish between these two mutations (Fig. 3). For this purpose, we used another restriction endonuclease, BsrI. The fifth nucleotide in the recognition sequence of BsrI is mutated in DNA harboring the W1197Xp mutation – ACTGA*A vs. ACTGGN (where * indicates the cutting site of BsrI) – whereas the mutation leading to P1199L has no effect on restriction by BsrI, as the mutation is located outside its recognition sequence. Restriction analysis with these two endonucleases therefore allows us to identify and to distinguish these two mutations unequivocally (Fig. 3C).

### Site-Directed Mutagenesis of Human Recombinant Somatic ACE

To confirm the mechanism by which the novel W1197X mutation might be responsible for the high plasma ACE level, we prepared several mutants of somatic ACE that harbor mutations near the stalk region. Table 1 demonstrates release/secretion characteristics of the generated mutants from CHO cells transfected with corresponding plasmids. The rate of ACE release was dramatically increased in each mutant ACE: whereas ACE activity released into the culture medium during 24 hrs cultivation was only 11% per day for wild-type ACE, ACE release increased to 49% per day for P1199L mutant and was more than 80% per day for the W1197X, S1211X and Q1230X mutants (Table 1). These data showed that all three ACE mutants lacking transmembrane domains are released into the culture medium practically completely, with minimal retention on the cell surface.

**Table 1:** Activity and rate of ACE release from CHO cells transfected with various ACEs.

ACE	ACE activity (mU/ml)		Rate of ACE release
	Soluble ACE	Membrane-bound	% per 24 h
Wild-type*	3.4±0.2	82.0±9.1	11.3±0.8
Wild-type#	0.06±0.2	2.3±0.1	7.8±0.4
Pro1199Leu#	6.4±0.4	29.7±2.1	48.9±5.1
Trp1197Stop#	12.0±0.4	7.7±2.1	83.5±2.0
Ser1211Stop#+	4.4±0.3	3.8±0.3	80.1±0.6
Gln1230StopX*&	628±39	195±10	89.8±2.1

CHO cells transfected (stable or transient) with cDNA of human recombinant wild-type ACE or indicated mutants were cultivated in 100 mm Petri dish in F12 medium with 10% FBS. Once cells become confluent, dishes were washed with PBS and a fresh Ultra CHO medium (10 ml) were added. After 24 hours of cultivation culture medium was collected, cells were washed with PBS and lysed with 3 ml HEPES buffer (20 mM, pH 7.5, NaCl 150 mM) with 8 mM CHAPS.

ACE activity was determined in culture medium (soluble ACE) and in a lysate (membrane-bound form) with Hip-His-Leu as a substrate and the rate of ACE shedding was calculated (ACE activity in culture medium X 100/ACE activity in culture medium + ACE activity in lysate).

* - Stable transfection:, # Transient transfection.

+ Plasmid with cDNA coding for this mutant was kindly provided by Dr. E. Sturrock (University of Cape Town, South Africa).

& Plasmid with cDNA coding for this mutant was kindly provided by Dr. F. Alhenc-Gelas (then INSERM U 367, Paris, France).

**Figure 5:**
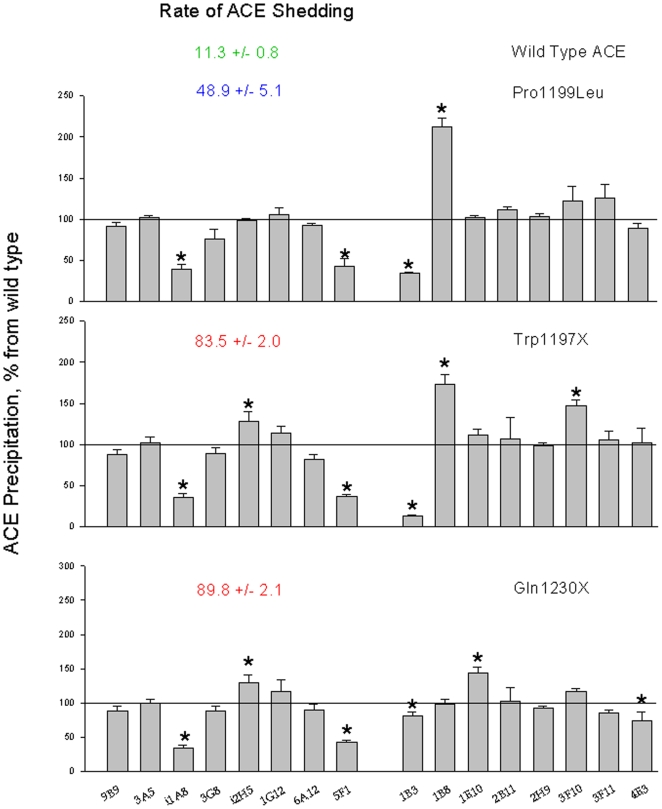
Conformational fingerprinting of mutated ACE with a set of mAbs to ACE. Data from the analysis of the precipitation of ACE activity from wild type recombinant human ACE, and recombinant mutant ACEs by 16 mAbs to human ACE were expressed as a ratio of ACE precipitation from Pro1199Leu mutant (A) or Trp1197Stop (B) to that for wild type ACE or ratio of ACE precipitation from Trp1197Stop to that from Pro1199Leu mutation (C). Data presented as a mean of three independent determinations (which were not differ more than 10%) for each ratio. * - p<0.05 in comparison with values for wild type ACE (A and B) or with Pro1199Leu mutant (C).

Immunological “conformational fingerprinting” of recombinant mutant ACEs using 16 mAbs to different epitopes located on the N- and C-domains (Fig. 5) confirms that changes in conformation of ACE from subject N1 were due to the W1197X mutation. Thus, mAb 1B3 recognizes an epitope on the C terminal part of the C-domain of somatic ACE and is very sensitive to the conformation of the C terminal end (39). We imagine that the much shortened C terminal end of the Pro1199Leu or Trp1197X mutants might unmask the epitope for 1B3, whereas the longer C terminal end as in the case of Gln1230X might shield the binding site. Besides changes in the immunoprecipitation of mutant Trp1197Stop ACE by mAbs 1B3, 1B8 and 3F10, which are directed to the region affected by this mutation, there are several others mAbs whose binding is also changed – i1A8, i2H5, 5F1 (see Fig. 5). Interestingly, these mAbs changed their binding also to other mutant, lacking a transmembrane anchor-Gln1230Stop. We think that some of the changes in pattern of ACE immunoprecipitation by 16 mAbs seen in mutants lacking transmembrane anchor are likely due to the changes in glycosylation pattern of mutated ACE. The time of traffic of mutated ACEs through endoplasmic reticulum is much shorter than a wild-type ACE [41]–[42], [29] and as a result the glycosylation pattern of mutated ACEs is different from wild-type ACE; this could result in differential binding of some mAbs (i1A8, i2H5, and 5F1), because glycosylation sites on Asn residues are included in their epitopes [40], [43]–[45].

### Western Blot Analysis of Mutated ACEs

**Figure 6:**
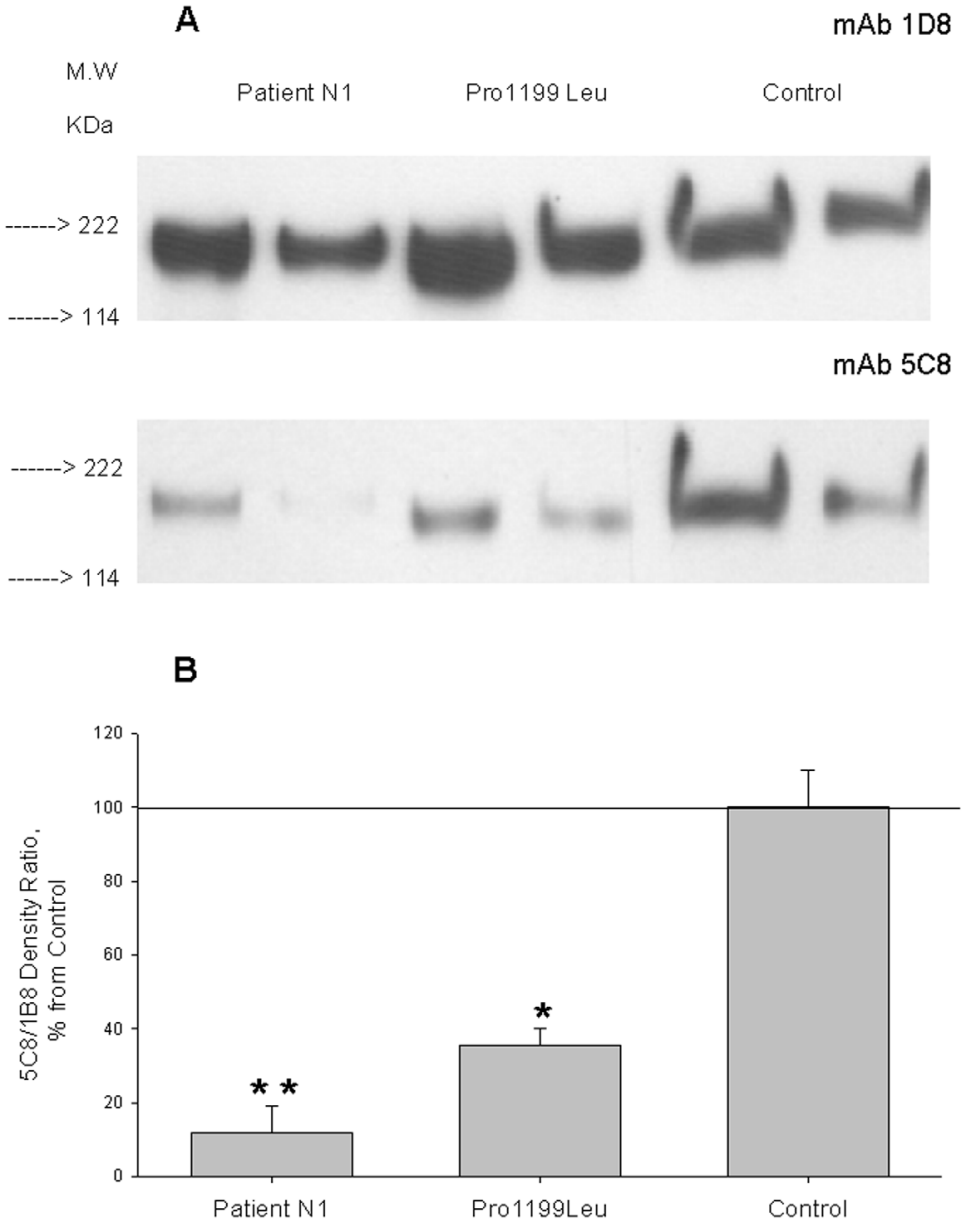
Western blot analysis of normal and mutant ACEs. **A**. ACE purified from plasma of patient N1, pooled plasma from 5 patients with hyper-ACE-emia (carriers of Pro1199Leu mutation) and pooled plasma from 5 healthy individuals with normal blood ACE level were equilibrated to 50 mU/ml of ACE activity (HHL as a substrate), boiled and 40 and 20 µl of each sample run in 4–15% radient gel in reducing conditions. Proteins transferred on PVDF-Plus membrane were revealed with 2 µg/ml of indicated mAb. Molecular weight is shown on the left. **B**. Revelation of blood ACE presented in panel A was quantified by densitometry of the bands. Data presented as a ratio of density with mAb 5C8 to that with mAb 1D8. Revelation of ACE from the blood of carriers of Pro1199Leu or Thr1197Stop (who contains a mixture of normal and mutated ACEs in their blood) by mAb 5C8 dramatically diminished.

We performed Western blotting of ACE purified from plasma of subject N1 using two mAbs to denatured human ACE: mAb 1D8, which recognizes a sequential epitope at the beginning of C domain [46], and mAb 5C8, which is directed to the C-terminal end of soluble ACE and of which binding was dramatically decreased to mutant P1199L ACE [47]. Binding of 5C8 was significantly abolished with both P1199L and W1197X mutants, whereas mAb 1D8 recognized similarly both wild-type and mutant ACE (Fig. 6A,B). Therefore, quantification of 5C8/1D8 binding ratio allowed us to identify the carriers of either mutation (but not to distinguish between them) using Western blotting of their blood ACE. The sensitivity of ACE detection with these mAbs allowed us to use even unprocessed blood from carriers of these mutations (not shown).

### Analysis of Ethnic Origin for the Mutated ACE Haplotype

To find human populations which might include other carriers of the W1197X mutation, we performed a haplotype-based analysis of the paternal (mutant) allele of subject N1's ACE gene. To reconstruct this haplotype, we sequenced her ACE genomic DNA and partially sequenced and genotyped both parents of N1. A panel of 12 SNPs specific for the mutated paternal ACE allele of N1 patient is shown in Table 2. To search for haplotype matches of N1, we used publicly available data on four populations of different ethnicity: Yorubans in Ibadan, Nigeria (YRI, n = 90); Japanese in Tokyo, Japan (JPT, n = 45); Han Chinese in Beijing, China (CHB, n = 45); and Utah residents with ancestry from northern and western Europe (Centre d'Etude du Polymorphisme Humain, CEPH) (CEU, n = 90). ACE genomic sequences in these populations were characterized with a set of 75 SNPs.

**Table 2:** Haplotype reconstruction of the paternal copy of ACE from patient N1.

Nomenclatures of the polymorphic markers of ACE gene*				
HapMap	Previous number [73]	Rieder's position [74]	Localization	Haplotype
rs4291	A-240 (-239)T	2400	Promoter	A
rs4292	T-93C	2547	Promoter	T
rs4309	T1237C	8125	Exon 8	C/T
rs4318	A7941G	10578	Exon 13	A
rs4331	G2215A	12257	Exon 15	A
rs4335	A10539G	NA	Intron 16	A
rs4340	Alu I/D	14095	Intron 16	D
rs4343	G2350A	14521	Exon 17	A
rs4353	A15990G	18912	Intron 20	A
rs4359	C17911T	20833	Intron 23	T
rs4362	NA	22251	Exon 24	T
rs4363	A20060G	22982	Intron 25	G

* Haplotype reconstruction of the paternal copy of the gene was based on sequencing of ACE from patient N1 and their parents. Novel mutation Trp1197Stop is located in exon 25 between SNPs rs4362 and rs4363. Selected 12 SNP are presented in the Table.

NA- not analyzed.

**Figure 7:**
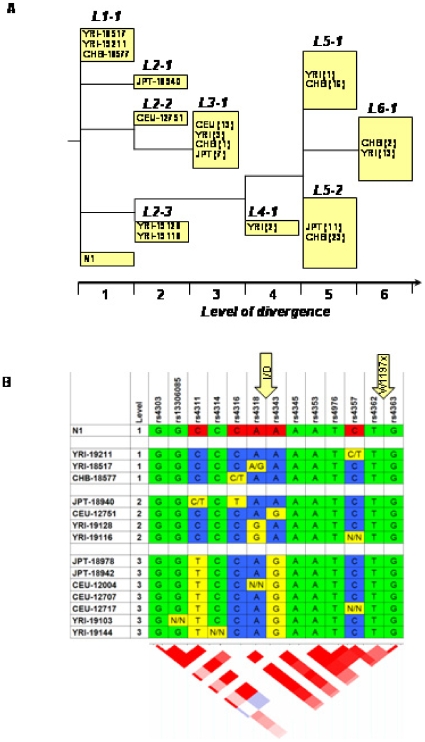
Hierarchical clustering of ACE haplotypes from different ethnic populations. **A**. Most parsimonious hierarchical classification tree for ACE haplotypes of individuals from four different ethnic origins and patient N1 is shown. Cladistic analysis was based on 13 informative polymorphisms and revealed 6 levels of divergence. Detailed description of samples is shown only for first two levels; for higher divergence only number of samples is indicated in parentheses. The size of boxes correlates with number of samples within the clade. Human populations were CEU, Caucasians; JPT, Japan; YRI, Africans; CHB, Chinese; and N1, patient with Trp1197Stop mutation. N1 patient is similar to Nigerians YRI-19211 and YRI-18517, and one Chinese CHB-18577. **B**. ACE genotypes and haplotypes for patient N1 and individuals from levels 1, 2 and 3 are shown. Positions highlighted in green were conservative in all shown individuals. Polymorphic nucleotides in N1 are highlighted in red, and show in alternative colors (yellow vs. blue) in other clades/individuals. Some positions were C/T or A/G heterozygous; or genotyping data were missing (N/N). SNPs (“rs” with number), position of the I/D polymorphism, and the position of W1197X mutation in N1 are shown on the top of the table. Linkage disequilibrium plot for Nigerian population is presented on the bottom (source: International HapMap project at http://www.hapmap.org).

First, from the set of 75 SNPs, 12 SNPs randomly distributed throughout the ACE gene and homozygous in subject N1 were chosen. Second, from the sets of individuals from these four populations (YRI, JPT, CHB and CEU), patients who were heterozygous for any of the 13 SNPs were excluded from further analysis. Finally, we excluded haplotypes with information missing for more than one chosen SNP. This selection procedure, which we consider to be random in regard to SNPs and individuals, yielded with data containing total of 100 individuals: n = 25 of CHB, n = 19 of JPT, n = 24 of YRI, and n = 32 of CEU population. This dataset was used for classification with PHYLIP; the most parsimonious tree is presented in Fig. 7 (A). We identified 9 clades, although number of samples within each clade varied. The haplotype of the N1's mutant ACE allele was found to be most similar to clade L1-1, which incorporates two Nigerians and one Chinese (YRI-19211, YRI-18517 and CHB-18577). Due to heterozygosity of some SNPs in the analyzed population, the haplotype of N1's mutant allele was found to be different from clade L1-1. However, N1 can be a perfect match with any of L1-1 members (Fig. 7B) and, therefore, N1 and clade L1-1 can be aggregated with high probability in a single clade.

### Assignment of the N1 Haplotype to Earlier Identified Clades

**Figure 8:**
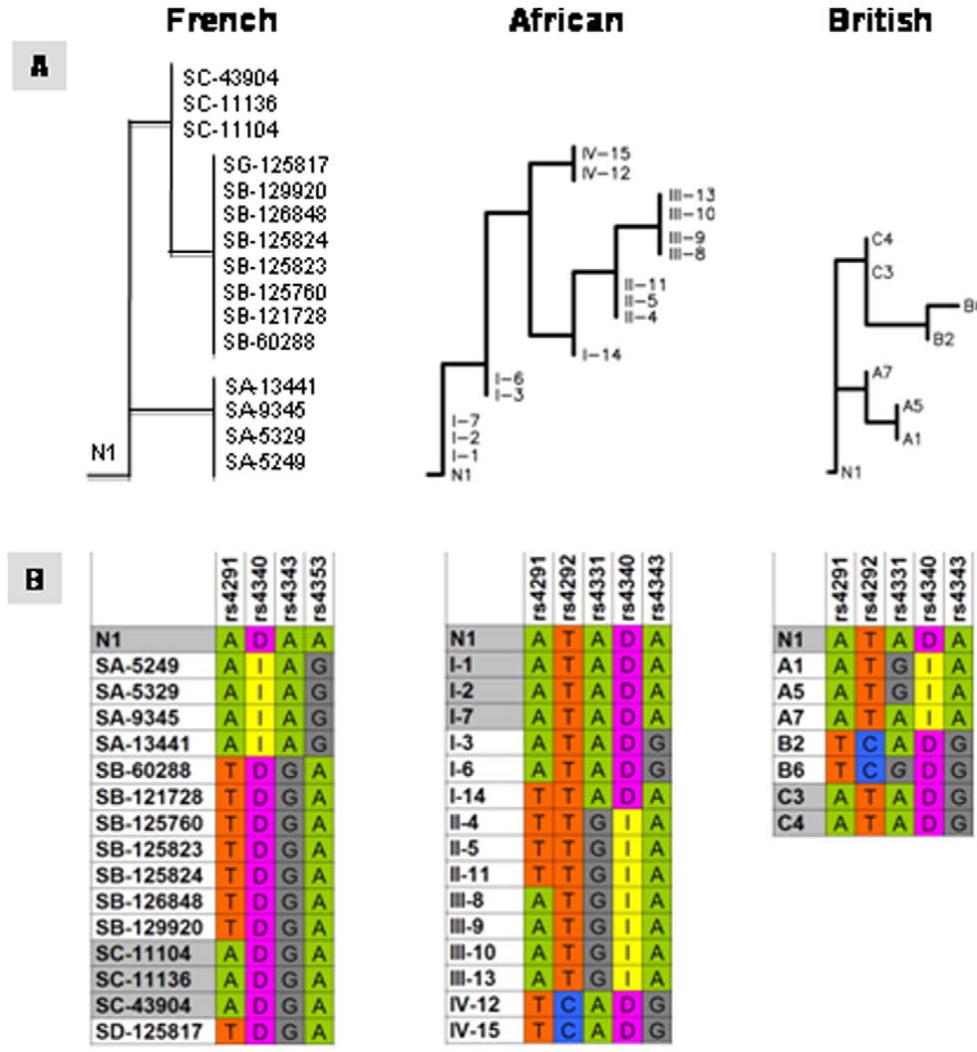
Clustering of the patient N1 haplotype into ACE clades. A. Clustering of the patient N1 haplotype along clades identified in French [51], British [48] and African-originated [50] populations. B. Detailed haplotypes of identified clades and N1 are presented. N1 carrier of the W1197X mutation perfectly matched with Africa-originated clade I (sub-clades 1, 2, and 7) and was consistently associated with clades represented high ACE plasma level (see text).

We performed further classification of the N1 haplotype in relation to earlier identified clades associated with high and low plasma ACE level using cladistic analysis. The following populations were analyzed: Caucasian British [48]–[49]; Nigerians, Jamaicans and African-American [50]; and French [51]. Since different set of SNPs were used in each study, we performed three separate analyses for N1 for every set of previously identified clades (Fig. 8). We found one perfect match in all three clade systems: N1 was assigned in the same clade with I-1, I-2, and I-7 clades of Yorubans from Nigeria (Fig. 8B). Patient N1 haplotype was most similar to clades SA-43904, SC-11136, and SC-11104 in the French population and to A7, C3 and C4 clades in the British population (Fig. 8B).

### Clinical Characterization of Subject N1 and Her Relatives

Subject N1 reported symptoms suggestive of asthma (cough, chest tightness, and wheezing aggravated by cold air, dust exposure, and upper respiratory infection), and her spirometry demonstrated mild airflow obstruction that improved slightly acutely following inhaled bronchodilator. Several additional first and second degree relatives reported doctor-diagnosed asthma; when measured, spirometry disclosed mild airflow obstruction in those with reported asthma, and no airflow obstruction in those who denied having asthma. N1's father (N2) had moderate airflow obstruction on spirometry but had smoked cigarettes. There was also a history of hypertension in N1's family, though subject N1 did not have hypertension herself. There was no consistent relationship between the presence of high serum ACE and clinical phenotype in N1's family.

## Discussion

Recently, a molecular mechanism underlying the marked (5-fold) familial increase of plasma ACE was discovered: P1199L substitution leads to more accessibility at the stalk region for ACE secretase and for the enhancement of the cleavage-secretion process [28]–[29]. We developed a convenient assay for the detection of this particular mutation based on analysis of the plasma ACE binding to mAb 1B3 (directed to the C-terminal part of soluble ACE) and binding to mAb 9B9 (on the N-terminal domain of ACE). The observed 1B3/9B9 binding ratio was 3-fold lower for affected individuals, thus allowing identification of this particular mutation in the stalk region of human somatic ACE with high sensitivity and specificity and without labor-time and cost-consuming sequencing or restriction analysis of the PCR products [39].

Here, we identified a family in whom a novel mutation – in which the codon TGG encoding Trp1197 is substituted in one allele by TGA (stop codon) – results in 13-fold elevation of ACE level in the blood. As a result of this mutation, half the ACE expressed in heterozygous individuals had a length of 1196 amino acids and lacked the transmembrane anchor, which starts from Val1228 [52]. Previous studies show that different ACE mutants that also lack the transmembrane anchor were directly secreted into the circulation [16], [53]. A similar mechanism accounts for subject N1's high serum ACE level.

The novel mutation carried by N1 can be detected using the above mentioned antibody-based assay (Figs. 1–2). However, although this approach identifies carriers of this new mutation (W1197X), it does not allow one to distinguish this from a previously described (P1199L) mutation. Therefore, we exploited restriction site polymorphisms in the DNA sequence encoding these two mutant isoforms (Fig. 3), implemented using PCR-amplified segments of DNA spanning these mutations.

The assays we developed – an antibody-based assay (1B3/9B9 ratio) and a restriction analysis of the PCR product – distinguish easily between elevation of blood ACE due to granulomatous diseases (sarcoidosis in particular) or Gaucher disease and hyper-ACE-emia due to lack of transmembrane domain, thus preventing false diagnosis and unnecessary treatment. Despite the fact that patients with the Pro1199Leu mutation exhibit no clinical abnormalities [28], the search for these mutations is of considerable clinical importance. Genetically determined elevation of ACE level may lead to false diagnosis of sarcoidosis and consequently to unnecessary long-term immunosuppresive treatment [30].

This mutation raises an important question – how much ACE (tissue and/or blood) is appropriate to maintain healthy conditions? Individuals without any functional ACE may die *in utero* or within 24 h of birth as a result of profound developmental abnormality of proximal kidney tubules – renal tubular dysgenesis [54]–[55]. In one family, a stop codon was introduced after Arg236 and in another family the correct sequence of ACE was through Pro402 - mature somatic ACE numbering [10]. Therefore, in both families affected homozygous individuals have no functional ACE. The same type of renal lesions was seen in fetuses that were exposed to ACE inhibitors [56]). Thus, it seems that for normal renal development it is necessary to have at least some minimum level of ACE, and as a consequence, a minimal level of Angiotensin II (Ang II) during development. Also, it is necessary to mention that mainly tissue ACE determines the conversion of Ang I to Ang II. When we found several Dutch families with the Pro1199Leu ACE mutation that leads to a 5-fold increase in blood ACE (due to increase in ACE shedding), we determined Ang I and And II levels in the affected subjects and in their unaffected relatives. Ang I level in patients with 5-fold elevated ACE tended to be only slightly (78%) lower than in controls, and this trend was not statistically significant. Ang II in the “high-ACE” group was just double that of the control group (5.0+5.0 versus 2.2+1.4 pmol/L in control group, p = 0.034) [28]. Furthermore, we would not expect to see a dramatic (much more than double) increase in the blood level of Ang II of affected family members because the levels of ACE in the tissues are more than an order of magnitude higher than those in the blood. Therefore, an overall conversion of Ang I to Ang II (per gram of any tissue) should not be dramatically increased in the affected members of this family.

We believe that despite the fact that tissue ACE in N1 patient should be just half of that of non-affected individuals, the huge excess of blood ACE in this individual perhaps compensates for a diminished level of tissue ACE. This possibility is supported by the finding of Bernstein's group, who demonstrated that the blood pressure of mice lacking tissue ACE, but having 80% of blood ACE, is indistinguishable from that of wild type mice [57]. Therefore, homeostasis can be maintained without the localized production of Ang II [58].

The demonstration of renal tubular dysgenesis in humans having no normal allele of ACE raises a serious question about the non-identity of mouse models and human pathologies. In mice a complete lack of ACE does not reproduce renal tubular dysgenesis; ACE knock-out mice are hypotensive and are unable to effectively concentrate urine. This functional renal defect accompanies structural kidney malformation typified by underdevelopment of renal medulla and papilla (59). However fetuses of ACE knockout mice did not die *in utero* and at birth the kidneys from ACE knockout mice are structurally indistinguishable from those of wild-type littermate controls [3]. Differences in the renal phenotype between humans and mice are not fully understood, though it has been suggested that differences in the timing of nephrogenesis explain the discrepancy [55], [60]. We think that dramatic differences in ACE activity in organs and blood of mice and human also might play a role: ACE activity in mouse blood and tissues is 10-fold more that in human. Therefore in human with absence of ACE [54], [60], the level of Ang II might fall below some level critical for normal kidney development.

Our data indicate that W1197X mutation is not a spontaneous event that occurred *de novo* in subject N1, but rather it was inherited from earlier generations. Therefore, we hypothesized that cladistic analysis will help us to identify distant relatives of subject N1, which would allow the identification of additional individuals with N1's phenotype. For this purpose, we analyzed human populations of four different ethnic/geographic origins with representative set of 12 SNPs. Haplotypes clustering did not resolve ethnic groups in four independent clades, although significant correlation of ACE haplotypes with geographical origin was demonstrated. Most remarkably, we found a perfect match for patient N1 ACE haplotype with two individuals from Nigeria. Taking into account that N1 is an African American, we hypothesize that mutation W1197X occurred at least several hundreds years ago in Africa in a grand ancestor of N1 and Nigerians YRI-18517 and YRI-19111. If this is true, there is a good chance to find other carriers of the mutation in Nigeria. However, similarity between haplotypes of N1 and Chinese CHB-18577 might be attributable either to common origin of these haplotypes further back in human history or to spontaneous re-creation of haplotype L1-1 in Africa and China due to evolution convergence.

In all three populations, cladistic analysis shows that N1 fits into “D” type of Alu-I/D polymorphism (Fig. 8B), the type, as it was shown earlier, correlates with higher plasma ACE level [49]. On a more detailed scale, when a greater number of SNPs was employed, N1's haplotype also clustered with clades representing higher plasma ACE level. For instance, in the French population, clade “C” (members SA-43904, SC-11136, and SC-11104) was associated with as much as 70% increase of z-score for ACE activity in plasma as compared to clade A [51] (Fig. 8B). In the British population, N1 haplotype was closest to Alu-D-type clade “C” (C3, C4) from study of Keavney et al [48]. Individuals from clade “C” have plasma ACE level 68% higher than patients from clade “A” [48].

In the African population, N1's haplotype was identical to three clades (I-1, I-2, I-7) found originally by Bourzekri with coauthors [50]. This supports the possibility that the Trp1197Stop mutation occurs with significant frequency in Nigeria. It was demonstrated that polymorphism rs4343 (ACE8 according to author's notation) was extremely highly associated (p<10^-17^) with elevated serum concentration of ACE. The best model to fit ACE polymorphisms (n = 6) and plasma level in all studied populations (Nigeria, Jamaica, and US) showed the highest association in Nigerians. ACE8 SNP alone was attributed to as much as 21% of the total ACE serum level variance in Nigerian individuals [50].

Taking together data on association of higher ACE sera level with Alu-D-type polymorphism and with downstream rs4343 SNP, we address the question on possible linkage disequilibrium in the area of ACE flanked with these two polymorphisms. Indeed, linkage disequilibrium (LD) analysis shows that recombinations in this area are rare (LD plot on the bottom of Fig. 7B). This implies that Alu-D-type might associate with higher ACE serum activity because of the tight linkage with functional polymorphisms, which belong to stalk domain of ACE. The novel Trp1197Stop mutation we found in subject N1 is one of the best examples of functional SNPs of this kind. Additionally, in the study of Bourzekri et al. [50], the highest association with high serum level of ACE was also demonstrated for the SNP located close to stalk region.

Thus, we can consider at least two different mechanisms for regulation of ACE activity in blood. One mechanism is reliant upon control of gene expression and genetically linked both to loci in promoter region of the gene and other loci in genome. A second way to control ACE level in blood is reliant on spontaneous alterations within stalk region of the gene/protein. The previously identified Pro1199Leu mutation [28] and the most recently discovered Trp1197Stop mutation (this study) are two examples of this mode of regulation of ACE activity.

Might very high level of blood ACE in subject N1 and in affected members of her family play a role in the mild airflow obstruction seen in affected individuals? There are plausible potential mechanisms by which elevated blood ACE might promote features of asthma pathology. Excessive accumulation of airway smooth muscle, due to hyperplasia and/or hypertrophy [61], is a common occurrence in asthmatic airways. Ang II, generated through hydrolysis of Ang I by ACE, stimulates vascular smooth muscle cell growth both *in vitro* [62] and *in vivo* [63]. Ang II promotes airway myocyte hypertrophy *in vitro* [64]. Perhaps high blood levels of Ang II (due to constitutively high level of blood ACE) might have a similar effect on airway myocytes in vivo. Ang II can also contract human bronchial rings [65]. Interestingly, increased plasma levels of Ang II have been reported in acute severe asthma [66]–[67], and iv infusion of Ang II in mild asthmatics can cause bronchoconstriction [66]. Furthermore, Ang II potentiates methacholine-induced bronchoconstriction [65]. Recently, it was also shown that locally generated Ang II causes bronchoconstriction in guinea pigs [68].

Another theoretical mechanism by which subject N1's mutation might have contributed to her airflow obstruction could relate to accelerated bradykinin (BK) metabolism. BK is another substrate for ACE, perhaps the “best” with the highest Km [1]–[2], and is a potent vasodilator that induces cough and bronchoconstriction in subjects with asthma [69]–[70]. Perhaps decreased ACE tissue levels increase local tissue concentration of BK and so promote asthma symptoms.

Additional implication of a potential role of ACE in asthma comes from analysis of an ACE insertion/deletion (I/D) polymorphism in different asthmatic populations. The homozygous deletion (DD) genotype is associated with minor elevation of serum ACE activity: individuals with this genotype have 60–70% more ACE in the blood than carriers of the II genotype and approximately 30–40% more than the whole population mean [33], [71]. In some but not all studies (reviewed in [72]), the DD genotype is associated with asthma. Although these findings might have suggested that the extremely high blood ACE levels in N1 and her family contribute to their asthma, in the limited population we studied we found no significant association between the presence of the W1197X mutation and asthma. Thus, if the mutation is at all contributory, it does not appear to be a dominant predisposing factor in N1's family.

In summary, we have identified a novel W1197X mutation that results in dramatic elevation of serum ACE, and which can occur in individuals of African American descent who have mild airflow obstruction. Because the constellation of airflow obstruction and elevated serum ACE in African American individuals is one that should raise the possibility of sarcoidosis to pulmonologists, it is important to recognize that mutations of the ACE gene that are not associated with disease – including the novel W1197X mutation reported here – can also lead to substantial blood ACE elevation. Thus, whenever very high serum ACE is found in the evaluation of potential sarcoidosis cases, we recommend that evaluation for the W1197X and P1199L mutations should be considered.
